# Prior Spontaneous or Induced Abortion Is a Risk Factor for Cervical Dysfunction in Pregnant Women: a Systematic Review and Meta-analysis

**DOI:** 10.1007/s43032-023-01170-7

**Published:** 2023-02-13

**Authors:** Julia J. Brittain, Stacey E. Wahl, Jerome F. Strauss, Roberto Romero, Hope M. Wolf, Katherine Murphy, John W. Cyrus, Timothy P. York

**Affiliations:** 1 Healthcare Studies, University of Richmond, Richmond, VA USA; 2 Health Sciences Library, Virginia Commonwealth University, Richmond, VA USA; 3 Department of Obstetrics and Gynecology, University of Pennsylvania, Philadelphia, PA USA; 4 Department of Obstetrics and Gynecology, Virginia Commonwealth University, Richmond, VA USA; 5 Perinatology Research Branch, Division of Obstetrics and Maternal-Fetal Medicine, Division of Intramural Research, US Department of Health and Human Services, Eunice Kennedy Shriver National Institute of Child Health and Human Development, Detroit, MI USA; 6 Department of Obstetrics and Gynecology, University of Michigan, Ann Arbor, MI USA; 7 Department of Epidemiology and Biostatistics, Michigan State University, East Lansing, MI USA; 8 Center for Molecular Medicine and Genetics, Wayne State University, Detroit, MI USA; 9 Detroit Medical Center, Detroit, MI USA; 10 Department of Human and Molecular Genetics, Virginia Commonwealth University, Richmond, VA USA; 11 School of Medicine, Virginia Commonwealth University, Richmond, VA USA

**Keywords:** Cervical length, Cervical insufficiency, Cervical incompetence, Abortion, Pregnancy, Preterm birth

## Abstract

A history of abortion is associated with cervical dysfunction during pregnancy, but there remains uncertainty about whether risk can be stratified by the abortion type, the abortion procedure, or number of previous abortions. The objective of this study was to verify the relationship between cervical dysfunction measures in pregnancies with and without a history of termination. Embase and Medline databases were searched from 01 January 1960 to 01 March 2022 resulting in a full-text review of 28 studies. The Newcastle–Ottawa Scale (NOS) was used to assess the quality and risk of bias for non-randomized studies. The meta-analysis consisted of 6 studies that met all inclusion and exclusion criteria and included a combined total of 2,513,044 pregnancies. Cervical dysfunction was defined as either cervical insufficiency/incompetence in 4 of the studies and as short cervix in the others. Results from a random-effects model using reported adjusted odds ratios (aOR) estimated an increase in the odds of 2.71 (95% CI 1.76, 4.16) for cervical dysfunction in the current pregnancy related to a history of induced or spontaneous abortion. Subgroup analyses with only induced abortions (surgical/medical) estimated an aOR of 2.54 (95% CI 1.41, 4.57), while studies limited to surgical abortions had an aOR of 4.08 (95% CI 2.84, 5.86). The risk of cervical dysfunction in the current pregnancy was also found to be dependent on the number of previous abortions. In this meta-analysis, a prior history of abortion, and specifically induced abortions, was associated with cervical dysfunction. The protocol was registered in PROSPERO (CRD42020209723).

## Introduction 

**Fig. 1:**
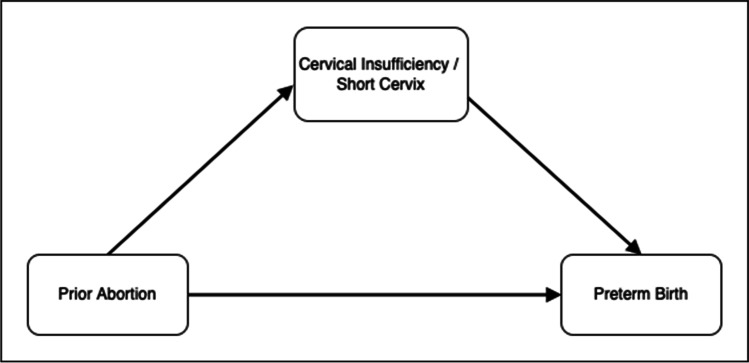
Conceptual model illustrating the mediation of cervical dysfunction on the relationship between prior abortion and preterm birth

The cervical insufficiency syndrome affects 1% of the obstetric population and is characterized by recurrent spontaneous preterm births and/or spontaneous abortions in the second trimester of pregnancy [1]. A short cervix measured by transvaginal ultrasound is used as a diagnostic criterion for cervical insufficiency [2] and for the prediction of preterm birth [3]. A history of spontaneous and induced abortions has been shown to correlate with both a short cervix/cervical insufficiency [4] and preterm birth [5, 6]. The possibility that the influence of a prior abortion on the gestational age at birth is mediated, in part, through an insufficient cervix has been suggested (Fig. 1) [5, 7].

Cervical insufficiency is defined by a transvaginal ultrasound cervical measurement of < 25 mm before 24 weeks gestational age in singleton pregnancies with a history of at least one pregnancy loss, preterm birth, or the presence of cervical changes detected by physical exam before 24 weeks [2, 8]. A diagnosis of cervical insufficiency has also been suggested by the presence of a short cervix and the observation of significant risk factors other than previous pregnancy loss [8]. While demographic factors have been associated with a short cervix [9, 10], there is limited understanding of pathophysiological mechanisms contributing to cervical dysfunction during pregnancy. One intriguing question is whether mechanical trauma to the cervical tissue or infection related to invasive procedures, such as cone biopsies and surgical and medical abortions abortions, may also contribute to risk for cervical dysfunction in subsequent pregnancies [7]. A better pathophysiological understanding of factors that may complicate the biomechanical transition the cervix undergoes between maintaining the fetus in the uterus to cervical remodeling leading to delivery is needed [11].

A short cervix is a risk factor for spontaneous preterm birth [4], while cervical insufficiency is a clinical diagnosis, but both reflect overlapping aspects of the condition of the cervix during pregnancy. For example, sonographic cervical length measurement approximates cervical effacement [12], a necessary condition for cervical ripening, and if occurring in the mid-trimester is a criterion for cervical insufficiency. Indeed, sonographic cervical length has been shown to indirectly measure cervical competence, thus providing a rationale for reconsidering this clinical diagnosis as a continuous trait [13]. Thus, the development of a short cervix and cervical insufficiency can both be influenced by congenital factors, loss of cervical tissue due to surgical procedures, intrauterine infection, and primary cervical disease [12]. Although both these measures cannot be equated, they provide an index of cervical health and potential complications during pregnancy.

The results of two related systematic reviews and meta-analyses that considers 49 unique studies conclude there is a significant relationship between a prior abortion and preterm birth [5, 6]. These studies are relevant considering the possible relationship between abortion, cervical dysfunction, and preterm birth (Fig. 1). More specifically, Lemmers et al. report an OR for preterm birth of 1.29 (95% CI 1.17, 1.42) for women with a history of dilatation and curettage compared to women absent a history of these procedures [5]. Saccone et al. report an OR for preterm birth of 1.44 (95% CI 1.09, 1.90) for women with a history of prior uterine evacuation versus women without a history [6]. Both studies stress the importance of conditional factors that could modify the primary relationship of interest and perform a series of sub-analyses to clarify clinical findings. Lemmers et al. report a slightly higher risk of preterm birth for a history of D&C compared to medically managed abortion (OR 1.19, 95% CI 1.10, 1.28). Saccone et al. report an OR for preterm birth of 1.52 (95% CI 1.08, 2.16) restricting to surgical induced abortions and an OR of 1.50 (95% CI 1.00, 2.25) when restricting to medically managed abortion. Both meta-analyses confirm an increasing risk of preterm birth with a history of multiple surgical induced abortions which supports a causal interpretation [5].

The aim of this systematic review and meta-analysis was to identify and report on the cumulative evidence for the relationship between a prior spontaneous or induced abortion and the risk of cervical dysfunction. Sub-analyses were conceived to test this relationship considering the type of abortion procedure, by induced or spontaneous abortions, based on the number previous abortions, and by definition of cervical health measure used by the study (i.e., short cervix or cervical insufficiency).

## Methods

### Search Strategy and Sources

The systematic review identified research studies published over the last 60 years, beginning in January of 1960 to 01 March 2022, to capture the span of time whereby both surgical and medical abortion procedures were widely adopted. Databases queried included Medline and Embase using the OVID interface. The search strategy for identifying qualified studies was developed using a controlled vocabulary and related keywords connecting to previous cervical trauma and cervical health. Initial concepts were developed in accordance with the Population, Exposure, Outcomes (PEO) framework [14], with assistance from a research librarian, and included female, human, pregnancy, abortion, induced/adverse effects, pregnancy reduction, multifetal pregnancy reduction, dilatation and curettage, loop electrosurgical procedure, dilatation and evacuation, endocervical curettage, cone biopsy, punch biopsy, spontaneous abortion, cervix uteri/pathology, uterine cervical, incompetence, ultrasonography, prenatal/methods, cervical, cervical insufficiency, cervical shortening, uterine cervical incompetence, and cervical length measurement. Initial concepts were translated for use in each database (MeSH for Medline and EmTree for Embase) and included related keywords. The PROSPERO repository was searched to identify other registered studies with a similar focus [15]. The Preferred Reporting Item for Systematic Reviews and Meta-Analysis (PRISMA) was used as a guide to report methods and results [16].

### Study Selection

Eligible studies included peer-reviewed publications and excluded dissertations, case studies, and unpublished literature. Eligibility was extended regardless of cohort location, nationality, or racial group identification or upon original publication language, if translated to English. Studies were included that investigated the relationship between previous abortion status and cervical insufficiency (formerly called cervical incompetence) during pregnancy as the primary outcome. The relationship between cervical insufficiency and prior history of cervical trauma was included to ensure that all eligible studies related to abortion and cervical health were identified. Eligible studies also included cervical length, as long as information regarding prior history of abortion and the relationship between both variables were reported. A short cervix was initially defined in pregnant women as a cervical length shorter than 25 mm between 18 and 24 weeks of gestation. The definitions of cervical insufficiency and cervical incompetence were evaluated within each study to assess homogeneity of results. An additional outcome included a dose–response relationship between the number of previous abortions and cervical insufficiency/shortening.

Prior abortion history was the primary exposure examined and included but was not limited to oral mifepristone (Mifeprex) and oral misoprostol (Cytotec), aspiration abortion, dilation and curettage (D&C), dilation and evacuation (D&E) abortion, and spontaneous abortion. Cervical procedures such as LEEP, endocervical curettage (ECC), cone biopsy, and punch biopsy were considered as indicators of possible cervical trauma. A pap smear was not considered as a cervical procedure, and multifetal pregnancy reductions were not considered as an eligible exposure.

### Data Extraction and Risk of Bias Assessment

Literature identified by database searches was annotated electronically by two independent reviewers. Variables collected included author and publication year, overall format of study, period of sample collection, location of research study, number of pregnancies included, definition of the cervical health measure, indication of abortion method, gestational age of abortion, presence of covariates in statistical models, and crude/adjusted effect size summary statistics. Any differences were resolved by a third reviewer. Study authors were contacted for missing information.

The quality of individual studies was evaluated using the Newcastle–Ottawa Scale (NOS) [17] by two independent reviewers. NOS is used for evaluating the quality of non-randomized studies in meta-analyses based across the domains of selection, comparability, and exposure. Each of the domains is divided into more specific categories, and each category, except comparability, can be awarded a maximum of one point. Each study could receive a maximum of 9 points, and studies that receive less than 5 points indicate a high risk of bias.

### Data Analysis

The meta-analysis was conducted using the R metafor package version 3.0–2 [18]. Models were fit separately using the effect sizes reported by the crude and/or adjusted odds ratios and associated standard errors, as applicable. Sampling variances were estimated by back-calculation of the 95% confidence intervals if required. Linear mixed-effects models were utilized when the test for heterogeneity using the Cochran’s *Q* test [19] was significant at *P* value of < 0.05; otherwise, a fixed effect model was considered. Random/mixed-effect models were specified using the restricted maximum-likelihood estimator and inverse variance weights. For the primary relationship of interest, the prediction interval, which presents the expected range of true effects in future studies, was reported [20]. A series of sensitivity analyses were performed to gauge the robustness of the primary result which tested the pooled association of a history of a previous abortion with cervical health during pregnancy. Planned sensitivity analyses were contingent on the breadth of studies identified from the systematic review.

## Results

### Study Selection

**Fig. 2:**
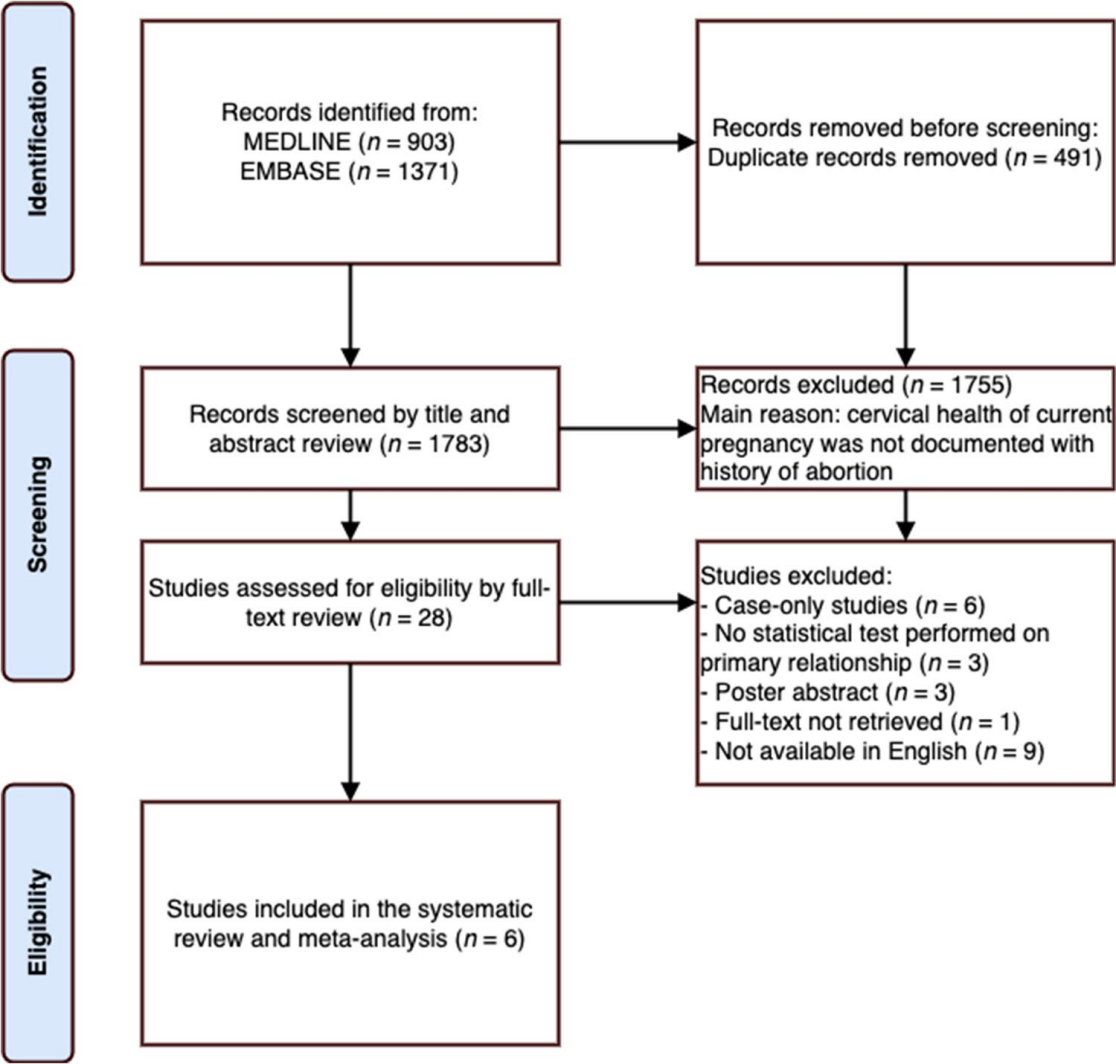
PRISMA flow diagram of search procedure and number of studies identified

The literature database search identified 2198 studies, of which 1715 were unique citations (Fig. 2). A total of 1687 studies were omitted after title and abstract screening since the majority did not contain cervical health measures in the current pregnancy and/or abortion history status. A full-text review was conducted on the 28 remaining studies, which identified 6 studies that met all criteria for the meta-analysis. Of the 22 articles excluded at this stage, 6 were case-only studies, 3 studies lacked a statistical analysis of the relationship of interest, 9 articles were not available in English, and the full text of 1 study was not retrievable. There were 3 poster abstracts identified, of which 2 corresponded to full-text articles present in this list. The retained studies included 2 prospective [9, 21] and 4 retrospective [7, 22–24] cohorts represented by 2,513,044 pregnancies (Table 1).

**Table 1:** Characteristics of the included studies on prior termination of pregnancy and cervical health

Author	Period	Location	Study type	Number of pregnancies assessed	Cervical health measure	Pregnancy abortion indicator	Gestational age at abortion	Covariates adjusted
Boelig (2018)	2012–2014	USA	Retrospective cohort	2672	Short cervix (≤ 20 mm) between 18–26 weeks 6 days	Prior uterine evacuation^*1*^	Any	Race, BMI, smoking status, prior full-term delivery
Tanner (2018)	2012	USA	Retrospective cohort	34,137	Cervical insufficiency	Previous pregnancy termination^*2*^	Not indicated	Maternal age, race/ethnic group, nulliparity, diabetes, hypertension, autoimmune disorders, any prior cervical procedure, any prior preterm delivery
Scholten (2013)	2000–2007	Netherlands	Retrospective cohort	1,357,894	Cervical incompetence treated by cerclage	Previous pregnancy termination^*3*^	Not indicated	Maternal age, ethnicity, socio-economic status, parity, smoking, drug dependence, pyelitis, polyhydramnios, history of spontaneous PTB, history of cervical incompetence or Shirodkar procedure, history of uterine myoma, or history of cervical surgery
Anum (2010)	2005	USA	Retrospective cohort	1,115,541	Cervical incompetence as indicated by birth certificates	Previous pregnancy termination^*4*^	Not indicated	Number of live births now living, number of live births now dead, history of previous preterm birth
Vyas (2006)	2003–2005	USA	Prospective case–control	98	Cervical insufficiency	Previous curettage procedure^*5*^	First trimester	Previous precipitous delivery, prolonged second stage of labor, interdelivery interval
Heath (1998)	1997–1998	UK	Prospective cohort	2702	Short cervix (≤ 15 mm) at 23 weeks	Previous pregnancy termination^*6*^	16–23 weeks	None

^*1*^Medical chart review indicating at least one D&C or D&E of a spontaneous (< 20 weeks) or induced abortion (any gestational age)

^*2*^Abstracted from electronic medical records system

^*3*^Based on self-report responses by the pregnant woman in a predefined pregnancy intake questionnaire. Surgical abortion was estimated for 90-95% of cases

^*4*^Recorded as a history of a single pregnancy termination from birth certificate records

^*5*^For the management of spontaneous abortion or voluntary termination of pregnancy

^*6*^Obstetric and medical history obtained from patients at their first antenatal visit

### Data Extraction and Quality Assessment

The included studies documented cervical health during pregnancy using variable but commonly accepted vocabulary which included cervical insufficiency, cervical incompetence, and cervical length. There were 2 studies that endorsed the outcome of interest as cervical insufficiency, as defined in one study using the International Classification of Diseases, Ninth Revision, Clinical Modification (ICD-9-CM) code [24], while the other study documented cases from a history of mid-trimester pregnancy loss with painless cervical dilation, no history of contraction, bleeding, or infection [21]. The latter study also tracked clinical symptoms to diagnose cervical insufficiency if a mid-trimester examination had complete cervical effacement or cervical dilation > 1 cm with protrusion of the membranes. Cervical incompetence was defined for subjects for those patients treated with cervical cerclage [22] and from US natality records indicating cervical incompetency [23]. The remaining studies utilized a definition of short cervix with slightly different thresholds including a cervix of ≤ 20 mm between 18 and ≤ 27 weeks [7] or a length ≤ 15 mm at 23 weeks [9].

There were 4 studies that defined the exposure of interest as a previous pregnancy termination, and these were documented by medical records from either the first antenatal visit [9], as at least a single pregnancy termination from birth certificate [23] or as self-reports from an intake questionnaire [22, 24]. No study was restricted to only medical abortion procedures, but surgical methods were primarily used in three studies where at least one D&C or D&E procedure was reported [7, 21] or from a cohort estimated to utilize surgical abortion in 90–95% of cases [22].

**Fig. 3:**
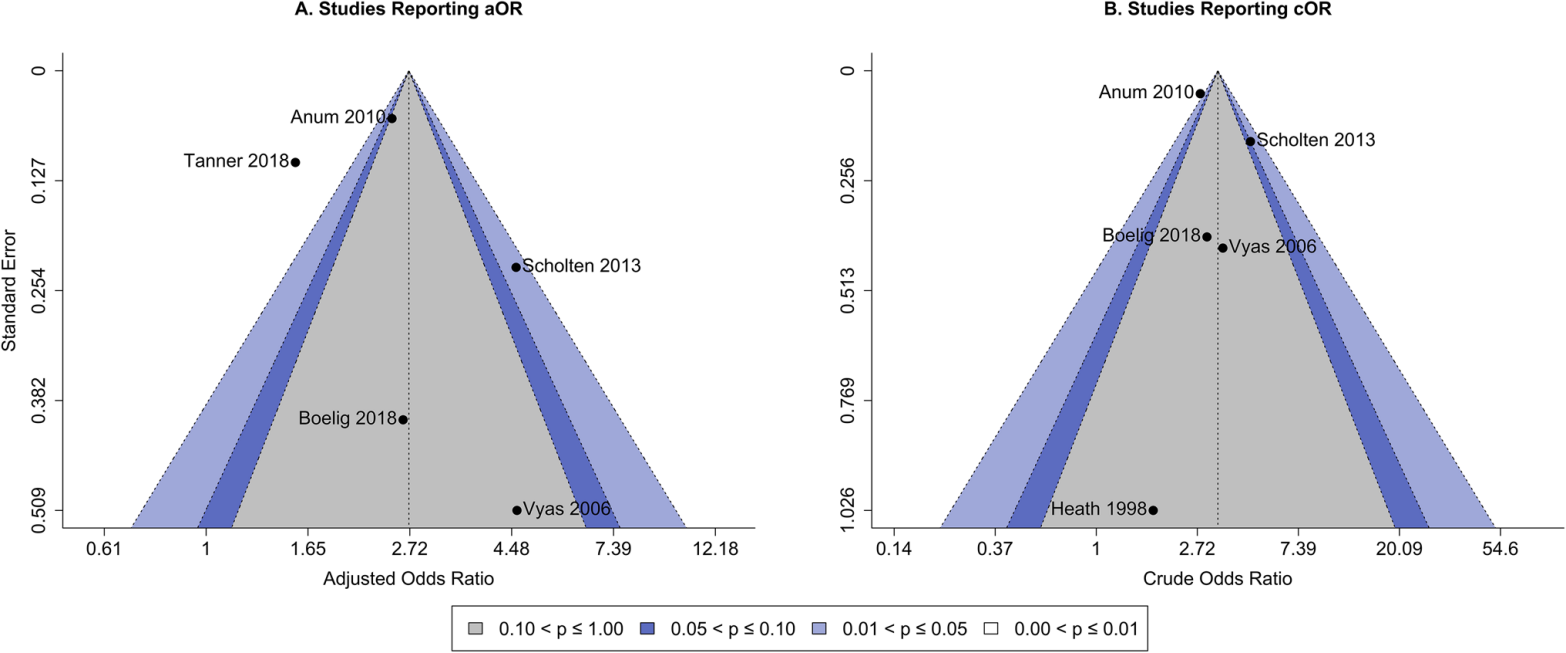
Funnel plot for observed study **A** adjusted and **B** crude odds ratios versus their standard errors. The vertical dashed line is the summary effect size and the shaded areas indicate the confidence interval regions. No asymmetry is visually observed nor an enrichment of studies within either significance contour indicating a lack of publication bias

A quality and bias assessment was conducted for each study using the Newcastle–Ottawa Scale (NOS) for cohort studies [17]. The NOS procedure assesses studies on 3 domains including (1) the selection of the cohort and ascertainment of the exposure; (2) on the comparability of cohorts by controlling for important factors and; and (3) by the assessment of the outcome. NOS scores are calculated by summing across domains with the maximum obtainable score being 9 where a score of ≥ 7 is generally considered to be of high quality. All retained studies provided sufficient methodological detail to describe the experimental design in each of these domains. The overall quality assessment was consistent across studies with a median NOS score of 8 and a range of 6 to 9. The Egger’s regression test for funnel plot asymmetry was not significant for either the adjusted OR (*β* = 1.14*, P* = 0.256) or crude OR (*β* =  − 0.26*, P* = 0.796). The individual study points in the respective funnel plots appeared symmetrical and evenly distributed across confidence contours indicating a lack of evidence for publication bias (Fig. 3).

### Meta-analysis

**Fig. 4:**
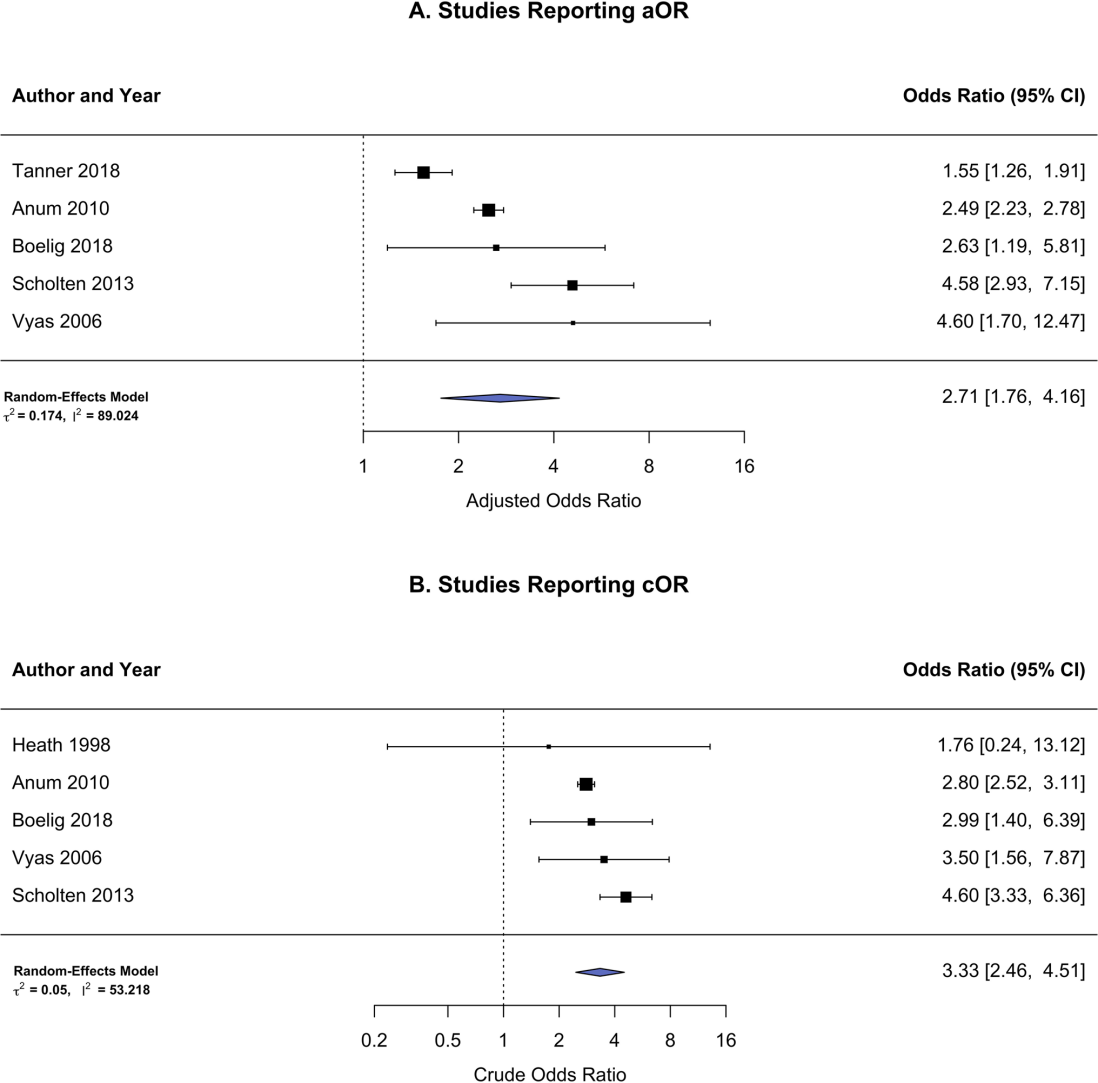
Forest plot for primary outcome risk (i.e., short cervix/cervical insufficiency) in women with a history of prior spontaneous or induced abortions. **A** Studies reporting adjusted odds ratios. **B** Studies reporting crude odds ratios

Results from a random-effects model using the reported adjusted odds ratios (aOR) estimated an increase in the odds of 2.71 (95% CI 1.76, 4.16) for cervical dysfunction in the current pregnancy related to a history of induced or spontaneous abortion and 3.33 (95% CI 2.46, 4.51) for unadjusted/crude odds ratios (cOR). The calculated 95% prediction interval for the adjusted model was 1.07 to 6.82 which is the expected range of true effects in future studies. A moderator analysis showed no differences in effect size due to NOS score (*β* =  − 0.21, 95% CI − 0.79–0.37, *P* = 0.475). Both the aOR and cOR were reported in four of six studies, while only the aOR was provided in Tanner et al., and only the cOR was reported in Heath et al. limiting a pooled analysis of all 6 studies. For these reasons, the complete set of analyses was reported separately for aOR and cOR statistics which included 2,510,342 and 2,478,907 recorded pregnancies, respectively (Fig. 4A–B). Model summaries for all meta-analyses are listed in Table 2.

**Table 2:** Summary of meta-analyses for risk of cervical dysfunction

		Model results		Heterogeneity			Prediction interval	
Exposure / control	Pregnancies	OR (95% CI)	*Z* val (*P* val)	Tau^2^ (SE)	*I*^2^	*H*^2^	Lower	Upper
Adjusted								
Prior abortion/no prior abortion	2,510,342	2.71 (1.76, 4.16)	4.53 (0.000)	0.174 (0.168)	89.02	9.11	1.07	6.82
Prior surgical abortion/no prior abortion	1,360,664	4.08 (2.84, 5.86)	7.61 (0.000)	0.000 (0.127)	0.00	1.00	2.84	5.86
Prior induced abortion/no prior abortion	2,507,572	2.54 (1.41, 4.57)	3.10 (0.002)	0.251 (0.272)	95.53	22.36	0.81	7.97
History of 1 abortion/no prior abortion	1,118,213	2.48 (2.23, 2.77)	16.56 (0.000)	0.000 (0.166)	0.00	1.00	2.23	2.77
History of 2 abortions/no prior abortion	1,118,213	4.64 (4.06, 5.31)	22.49 (0.000)	0.000 (0.218)	0.00	1.00	4.06	5.31
Prior abortion: cervical incompetence/no prior abortion	2,507,670	2.76 (1.64, 4.65)	3.81 (0.000)	0.229 (0.230)	93.08	14.44	0.94	8.07
Crude								
Prior abortion/no prior abortion	2,478,907	3.33 (2.46, 4.51)	7.81 (0.000)	0.050 (0.079)	53.22	2.14	1.95	5.68
Prior surgical abortion/no prior abortion	1,360,664	4.20 (3.18, 5.56)	10.07 (0.000)	0.000 (0.092)	0.00	1.00	3.18	5.56
Prior induced abortion/no prior abortion	2,476,137	3.38 (2.16, 5.29)	5.34 (0.000)	0.095 (0.155)	76.48	4.25	1.59	7.19
History of 1 abortion/no prior abortion	1,118,213	2.79 (2.52, 3.10)	19.34 (0.000)	0.000 (0.160)	0.00	1.00	2.52	3.10
History of 2 abortions/no prior abortion	1,118,213	6.00 (5.28, 6.83)	27.28 (0.000)	0.000 (0.204)	0.00	1.00	5.28	6.83
Prior abortion: cervical incompetence/no prior abortion	2,473,533	3.47 (2.40, 5.02)	6.60 (0.000)	0.070 (0.109)	72.89	3.69	1.84	6.55
Prior abortion: short cervix/no prior abortion	5374	2.80 (1.37, 5.70)	2.84 (0.005)	0.000 (0.850)	0.00	1.00	1.37	5.70

**Fig. 5:**
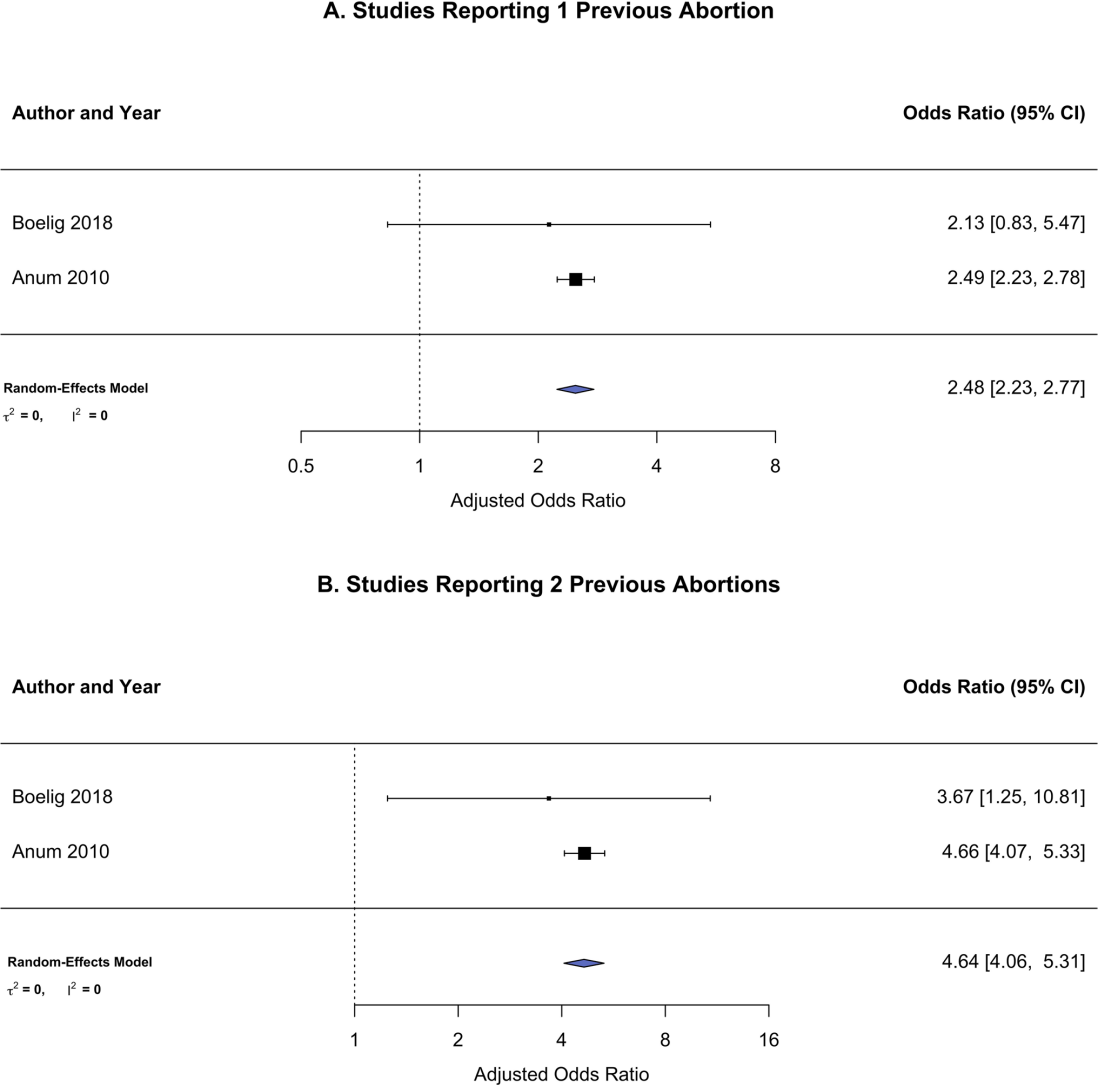
Forest plot for primary outcome risk (i.e., short cervix/cervical insufficiency) stratified by number of prior spontaneous or induced abortions. **A** Women reporting a history of 1 previous abortion. **B** Women reporting a history of 2 previous abortion

There were two published studies that both demonstrate a dose–response relationship by comparing women without a history of abortion to women with multiple abortions from a combined total of 1,118,213 pregnancies. In one study [23], increasing odds for cervical incompetence was seen after one abortion (aOR 2.49, 95% CI 2.23–2.77), after two abortions (aOR 4.66, 95% CI 4.07–5.33), after three abortions (aOR 8.07, 95% CI 6.77–9.61), and for four or more abortions (aOR 12.36, 95% CI 10.19–15.00). The other study [7] reports an increase in odds after one abortion (aOR 2.13, 95% CI 0.83–5.48), after two abortions (aOR 3.67, 95% CI 1.25–10.84), and for two or more abortions (aOR 3.52, 95% CI 1.33–9.33). The pooled analysis combined levels in common used by both studies and was found to be dependent on the number of previous abortions which increased from an aOR of 2.48 (95% CI 2.23, 2.77) for one reported abortion to an aOR of 4.64 (95% CI 4.06, 5.31) for two reported abortions (Fig. 5).

**Fig. 6:**
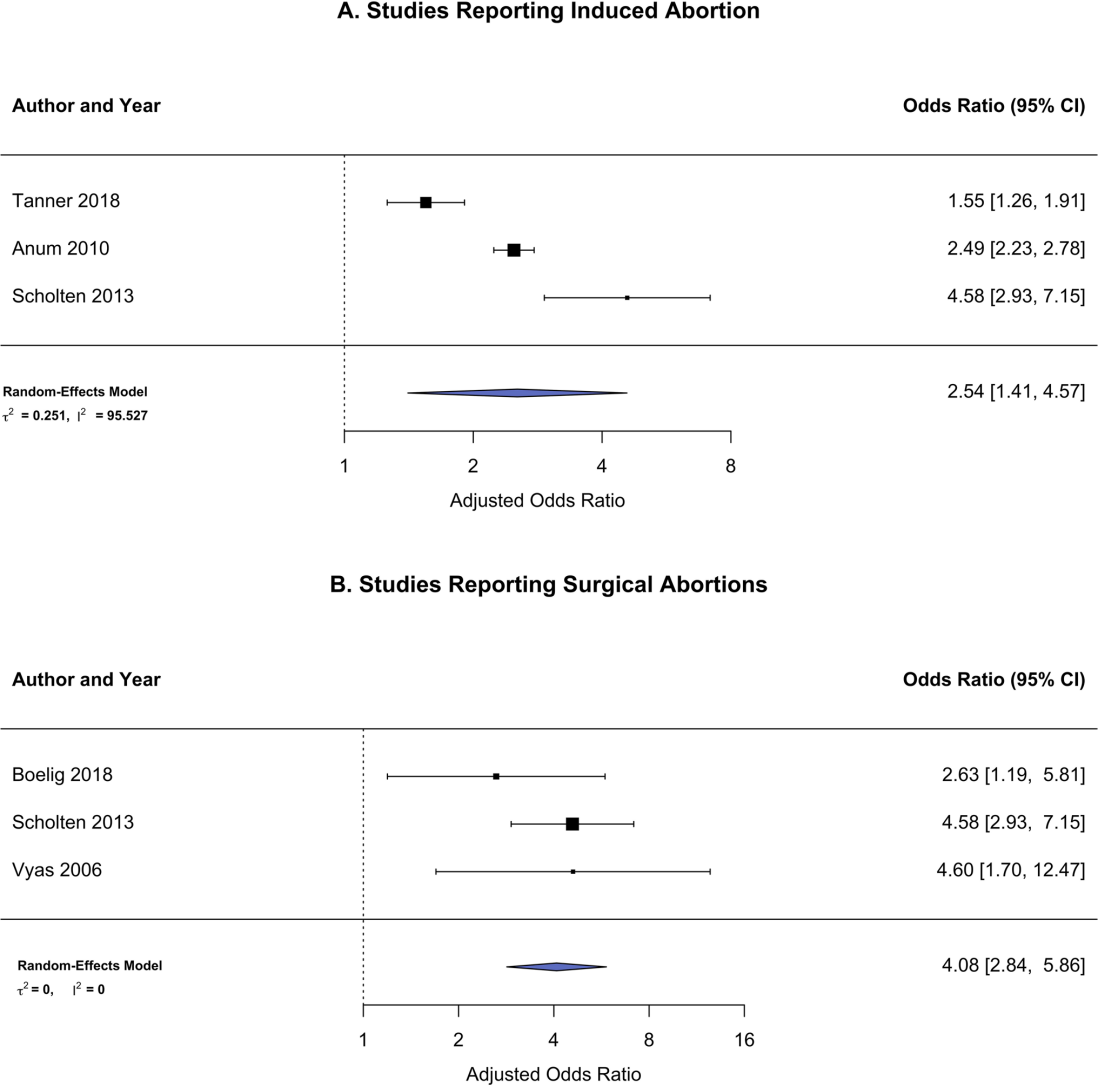
Forest plot for primary outcome risk (i.e., short cervix/cervical insufficiency) in women with a history of prior **A** induced abortions or **B** surgical abortions

There were four studies [9, 22–24] that include only induced abortions and show a pooled increased odds for cervical dysfunction in the current pregnancy: aOR 2.54 (95% CI 1.41, 4.57) (Fig. 6A). In the study by Heath et al., induced abortions are classified as distinct from miscarriages. The study by Anum et al. include abortions that are likely all induced as typically listed in US natality records and verified by personal communication with the author. In the large Netherlands Prenatal Registry (NPR) accessed by Scholten et al. study, different terminologies are used for pregnancy terminations and miscarriages. Finally, Tanner et al. indicate abortions as prior pregnancy terminations. Surgical abortion methods could be inferred from three of the retained studies [7, 21, 22], and pooled analysis showed an aOR of 4.08 (95% CI 2.84, 5.86) with cervical health complications (Fig. 6B). Scholten et al. restricted their investigation to pregnancy records from January 2000 to December 2007 to mitigate bias since medical terminations were not commonly performed during this time. The studies by Boelig et al. and Vyas et al. select only pregnancies with a history of operative abortion procedures.

**Fig. 7:**
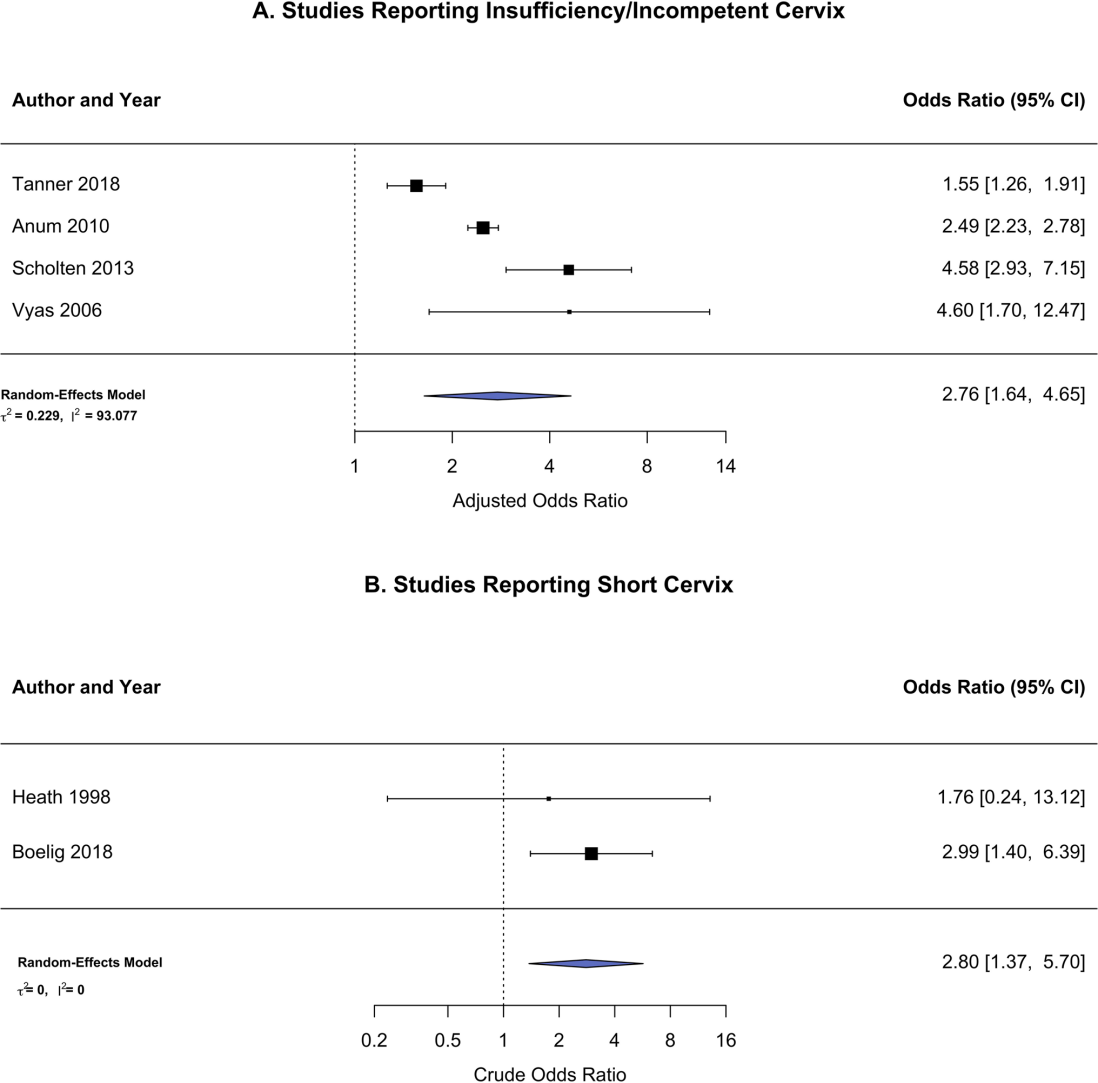
Forest plot for primary outcome risk in women with a history of prior spontaneous or induced abortions reported as either **A** short cervix/cervical insufficiency or **B** short cervix

Previous abortion was found to have increased odds for the four studies with the outcome defined as cervical insufficiency/incompetence (aOR 2.76, 95% CI 1.64, 4.65) and an increased odds for the two studies with short cervix as the endpoint (cOR 2.80, 95% CI 1.37, 5.70) (Fig. 7A–B). A moderator analysis showed no differences in effect size due to the definition of cervical health (*β* =  − 0.44, 95% CI − 1.38–1.28, *P* = 0.943).

## Discussion

### Summary of Main Findings

The results of the systematic review and meta-analysis confirmed that pregnant women with a history of a spontaneous or induced abortion, compared to those without, were at 2.71 times increased risk for cervical dysfunction during pregnancy, defined as either short cervix or cervical insufficiency/incompetence. Women reporting a single prior abortion had significantly higher risk for cervical dysfunction compared to women without a history of abortion. This risk was nearly twice as high for women who reported having two previous abortions compared to women without a history of abortion (OR = 2.48 versus 4.64), suggesting a dose–response relationship.

Sensitivity analyses restricted to studies that report only induced abortions revealed a higher risk of cervical dysfunction (OR = 2.54). While no study reported data from only medical abortions, those studies where surgical-only abortions were reported indicated a strong relationship between abortion and cervical dysfunction (OR = 4.08). The increased risk observed was similar whether the outcome was recorded as either short cervix or cervical insufficiency (OR = 2.80 and 2.76, respectively), and the results of a moderator analysis did not find evidence that these relationships differed by the definition of cervical health used.

### Interpretation of Findings and Clinical Implications

These findings are congruent with those from two recent systematic reviews and meta-analyses directed at evaluating the effect of prior abortion and risk for preterm birth [5, 6]. Namely, these studies are characterized by a heightened risk of preterm birth with a history of abortion procedures and an observed dose–response relationship. Interestingly, the odds ratio reported for the relationship between prior abortion and cervical dysfunction in this meta-analysis (OR = 2.71) was nearly twice as high as those reported for in Lemmers et al. (OR = 1.29) and Saccone et al. (OR = 1.44) that tested the relationship between preterm birth and prior dilatation and curettage or uterine evacuation, respectively. All three meta-analyses demonstrate an increased risk of reproductive health complications with increased number of prior abortions. Specifically, the dose–response relationship of prior abortion with both cervical dysfunction and preterm birth supports a causal interpretation [5] and the larger effect of prior abortion on cervical dysfunction versus preterm birth potentially positions cervical dysfunction as a mediating variable.

The closed cervix functions during pregnancy to support the developing fetus and as a barrier to protect the intrauterine environment from external pathogens [25]. For these reasons, the length of the cervix in the second trimester likely serves as one of the strongest predictors of preterm birth risk in the current pregnancy [4, 26]. The robust association of prior abortion with both cervical dysfunction and preterm birth provides a common cause to explain this observed risk. While a prior abortion could have other consequences for reproductive tissue, for instance, curettage damage of the endometrial tissue [5, 27], collectively, these findings are consistent with the conceptual model presented in Fig. 1. Considering the strong prima facie case for the interrelation among these clinical measures, a mediational analysis conducted from prospectively collected epidemiological data can test the extent in which changes in cervical tissue/length during pregnancy can account for the association between prior abortion and preterm birth [28]. Such an analysis would provide valuable insight into the pathophysiological mechanism behind the association between prior abortion and preterm birth.

The structural integrity of the cervix, determined by the cellular components and extracellular matrix network, is essential for carrying a pregnancy to term [29, 30]. The remodeling of the cervical stroma is a complex process that begins early in pregnancy and is necessary to transition from physically maintaining the pregnancy in the uterus to allowing the baby passage through the cervical canal during labor through cervical softening, effacement, and dilation [3]. In contrast to normal labor where the cervix is dilated slowly, the more rapid mechanical stretching of the cervix required by surgical abortion and later-term medical abortions can result in long-term cervical damage [5, 31]. The full scope of how surgical and medical abortion-driven complications affect the balance of cervical function in subsequent pregnancies is not well understood and may require an engineering framework to assess how the presence of an abnormal material structure of the cervix and/or abnormal anatomical changes throughout pregnancy can lead to birth complications [11]. A candidate mechanism could be related to disrupted changes in turnover of the extracellular matrix composition [30, 32, 33], but whether this could be due to cervical trauma has yet to be investigated.

Besides a history of multiple abortions, the next largest risk estimated in this meta-analysis was for studies that reported on surgical-only abortions. Whether an argument could be made that medical management would present a safer alternative to surgical methods is not possible from the data collected from this systematic review. Relative to increased preterm birth risk, the meta-analysis by Lemmers et al. show only a slight increase due to surgical versus medical management (OR = 1.19, 95% CI 1.10, 1.28), but critically no comparison could be made between women with prior medical management of abortion compared to women without a history of abortion. The meta-analysis of Saccone et al. was able to make this comparison, and results show a borderline significant risk due to medical management (OR = 1.50, 95% CI 1.00, 2.25) yet at the same order of magnitude as surgical methods from the same meta-analysis (OR = 1.45, 95% CI 1.27, 1.65). More studies are needed to evaluate the true risk of medical management considering this estimate was based on two relatively small studies compared to their estimate of surgical methods based on 27 studies, many of which contain sample sizes several orders of magnitude greater.

The increased risk of cervical dysfunction due to a history of abortion, and especially multiple abortions, raises the question of whether more intensive cervical health surveillance in pregnancy is warranted to initiate early interventions. Future research is needed to evaluate the scope of possible assessments of cervical health but may include the monitoring of intra-amniotic inflammation [34, 35], infection status [36–38], or less widely used modalities such as elastography [39, 40]. Enhanced monitoring of cervical length by serial measurements across pregnancy has been shown to more accurately predict preterm birth in twin gestations [41, 42] but not in singleton pregnancies [43].

### Limitations and Strengths

To the best of our knowledge, this is the first systematic review and meta-analysis of cervical dysfunction risk relative to prior abortion status. The limited number of studies meeting the systematic review selection criteria represents the state of research in this field, contains low levels of bias according to the NOS guideline, and includes over 2.5 million pregnancies assessed primarily from two studies [22, 23]. Studies identified are from Western European countries and the USA and include only prospective and retrospective cohorts which may limit the relevance of study conclusions to certain populations. For example, population-specific genetic factors may have influenced outcomes [44, 45]. While the search strategy was comprehensive and the calculated prediction intervals do not suggest additional studies would alter the conclusions, there is always a possibility that relevant studies were not identified.

The estimated risk of cervical dysfunction during pregnancy associated with prior abortion was nearly twice as large compared to contemporary meta-analyses where preterm birth was the outcome [5, 6]. Similar to these studies, data on medical abortion was limited, and sensitivity analyses were contingent on the available data. Although the cervical dysfunction outcome differed slightly among studies, they were clinically related, and moderation analysis did not show differences in association by definition used.

All but one study considered covariates in analyses [9], yet for those that did, the set of variables differed widely across studies. One study only reported adjusted effects [24]. For this reason, both the crude and adjusted odds ratios were reported as applicable, although adjusted values were preferred, and results show only small differences between estimate types. The testing of the dose–response effect was limited to a history of one and two prior abortions since only these frequencies were common across studies that considered this relationship.

## Conclusion

In comparison to recent systematic reviews and meta-analyses, the risk of cervical dysfunction was 1.9–2.1 times higher than the risk of preterm birth [5, 6] in women with a history of abortion compared to women without a history. The consistency of effects, the observed dose–response relationship, pathophysiological plausibility, and temporal ordering of measures supports a causal explanation for the relationship between prior abortion and cervical health dysfunction. The possibility that cervical health dysfunction serves as a mediating variable to, in part, account for the relationship between prior abortion and preterm birth is intriguing and should be explored in future studies. The lack of data on the medical management of abortion in this study and the unclear picture of similar effects in the other meta-analyses mentioned preclude a full assessment of the risk of this procedure. This study provides an updated assessment of the risk associated with prior abortion on cervical health that practitioners should review with women of reproductive age.

## Data Availability

Data available on request.
